# SvAnna: efficient and accurate pathogenicity prediction of coding and regulatory structural variants in long-read genome sequencing

**DOI:** 10.1186/s13073-022-01046-6

**Published:** 2022-04-28

**Authors:** Daniel Danis, Julius O. B. Jacobsen, Parithi Balachandran, Qihui Zhu, Feyza Yilmaz, Justin Reese, Matthias Haimel, Gholson J. Lyon, Ingo Helbig, Christopher J. Mungall, Christine R. Beck, Charles Lee, Damian Smedley, Peter N. Robinson

**Affiliations:** 1 The Jackson Laboratory for Genomic Medicine, Farmington, CT 06032 USA; 2 William Harvey Research Institute, Charterhouse Square, Barts and the London School of Medicine and Dentistry, Queen Mary University of London, London, EC1M 6BQ UK; 3 Division of Environmental Genomics and Systems Biology, Lawrence Berkeley National Laboratory, Berkeley, CA 94720 USA; 4 Ludwig Boltzmann Institute for Rare and Undiagnosed Diseases, Vienna, Austria; 5 St. Anna Children’s Cancer Research Institute, Vienna, Austria; 6 CeMM Research Center for Molecular Medicine of the Austrian Academy of Sciences, Vienna, Austria; 7 Present address: Global Computational Biology and Digital Sciences, Boehringer Ingelheim Regional Center Vienna GmbH & Co KG, 1120 Vienna, Austria; 8 Department of Human Genetics, New York State Institute for Basic Research in Developmental Disabilities, Staten Island, New York, USA; 9 Biology PhD Program, The Graduate Center, The City University of New York, New York, USA; 10 Division of Neurology, Children’s Hospital of Philadelphia, Philadelphia, PA USA; 11 The Epilepsy NeuroGenetics Initiative (ENGIN), Children’s Hospital of Philadelphia, Philadelphia, PA USA; 12 Department of Biomedical and Health Informatics (DBHi), Children’s Hospital of Philadelphia, Philadelphia, PA USA; 13 Department of Neurology, University of Pennsylvania, Philadelphia, PA USA; 14 Department of Genetics and Genome Sciences, University of Connecticut Health Center, Farmington, CT 06032 USA; 15 Institute for Systems Genomics, University of Connecticut, Storrs, CT 06269 USA

**Keywords:** Long-read sequencing, Whole genome sequencing, Structural variant

## Abstract

Structural variants (SVs) are implicated in the etiology of Mendelian diseases but have been systematically underascertained owing to sequencing technology limitations. Long-read sequencing enables comprehensive detection of SVs, but approaches for prioritization of candidate SVs are needed. Structural variant Annotation and analysis (SvAnna) assesses all classes of SVs and their intersection with transcripts and regulatory sequences, relating predicted effects on gene function with clinical phenotype data. SvAnna places 87% of deleterious SVs in the top ten ranks. The interpretable prioritizations offered by SvAnna will facilitate the widespread adoption of long-read sequencing in diagnostic genomics. SvAnna is available at https://github.com/TheJacksonLaboratory/SvAnna.

**Supplementary Information:**

The online version contains supplementary material available at 10.1186/s13073-022-01046-6.

## Background

Structural variants (SVs) range from 50 base pairs (bp) to megabases in size and can be classified into a wide range of events including deletions, tandem and interspersed duplications, insertions, inversions, translocations, or complex combinations of these events [1]. The advent of short-read exome sequencing in 2010 ushered in a decade of novel discoveries in Mendelian genetics and led to the introduction of diagnostic exome and subsequently genome sequencing. Over 100 short read-based mappers and over 40 short-read variant callers have been introduced since 2010; while performance has been steadily increasing, SV calling from short reads is reported to have a recall of between 10 and 70% associated with high false-positive rates [2, 3]. Long-read sequencing (LRS), including both PacBio single-molecule real-time sequencing (SMRT) and Oxford Nanopore sequencing, produces longer reads that can be more accurately mapped to the reference genome even in regions that are inaccessible to short-read sequencing (SRS) [4, 5].

LRS technology can address some of the shortcomings of SRS and enable more comprehensive detection of a broader range of SVs. A recent study with LRS in conjunction with additional methods such as single-cell template strand sequencing estimated that while 78% of SVs identified by SRS are concordant with LRS SV calls, only 30% of LRS SVs were observed in the short-read WGS callset; on average, 24,653 SVs were detected per genome by LRS [6]. SRS captures the majority of SVs affecting coding sequence in genes with existing evidence for dominant-acting pathogenic mutations from OMIM, and the majority of SVs identified only by LRS are located in highly repetitive regions that have been previously inaccessible to human disease studies [7]. Initial studies have appeared on LRS as a diagnostic tool for the diagnosis of Mendelian disease, with reports on the detection of large deletions, insertions, translocations, and tandem repeat expansions [4, 8–11]. LRS can be used to address cases in which SRS and sometimes cytogenetic or chromosomal microarray analysis has failed to identify an etiology, and therefore analysis may focus on intermediate size SVs (50 bp to 2 kb) difficult to detect with the other methods [12]. The community, however, lacks dedicated tools for prioritization of all classes of SVs identified by LRS experiments, which hinders the utility of LRS in Mendelian disease studies.

In this work, we introduce Structural variant Annotation and analysis (SvAnna), an integrated tool for the annotation and prioritization of SVs called in LRS data starting from variant call format (VCF) files produced by LRS SV callers such as pbsv, sniffles [13], and SVIM [14]. SvAnna is freely available for academic use (https://github.com/TheJacksonLaboratory/SvAnna) [15]. SvAnna prioritizes variants in light of their overlap with structural elements of genes and promoters. On a curated set of 182 case reports of 188 SVs underlying Mendelian disease, SvAnna placed the correct variant within the top 10 ranks (out of 62,337–107,233 variants per VCF file) in 87% of cases.

## Methods

### Comprehensive and harmonized representation of variants in VCF files

SvAnna was designed to capture all classes of structural variation represented in VCF files. VCF specification allows three notations for storing variant coordinates, alleles, and attributes. The variant coordinates and variant alleles can be specified using (a) default (sequence) representation (the ALT sequence is known, the variant end position is inferred from length of the ALT sequence), (b) symbolic representation (the ALT sequence is not provided, e.g., <DUP>, <INV>, the end position is reported in the INFO field), or (c) breakend notation for complex rearrangements, where adjacencies of the novel rearrangement are described on multiple lines using the square bracket notation. We developed a Java library called Svart [16] that decodes all three notations and provides a consistent API for all variant categories. Besides modeling variants, Svart standardizes representation of genomic elements, such as genes, transcripts, enhancers, repetitive regions, and dosage-sensitive regions, and handles conversion of coordinates between genomic coordinate systems and strands, and simplifies calculation of distances and overlaps between the genomic elements. SvAnna leverages Svart to represent structural variants specified in any valid VCF notation from both short-read and long-read VCF files in a harmonized form for the analysis of overlap of SVs with transcripts. For VCF records formatted in the breakend notation (BND), SvAnna assumes that the record represents a novel adjacency between the two contigs. The adjacencies are analyzed individually; SvAnna does not group the adjacencies based on the EVENT INFO field.

### Data sources

The input variants are filtered to remove the common SVs before the prioritization. SvAnna uses several sources of common SVs and their frequencies: Database of Genomic Variants [17], GnomAD SV [18], Human Genome Structural Variation Consortium (HGSVC) SVs freeze 3 [6], and dbSNP v151 databases (all accessed in April 2021). Transcript definitions were generated for RefSeq and Ensembl using Jannovar [19]. Enhancer definitions were extracted from the VISTA database [20]. Locations of repetitive elements were taken from the UCSC Genome Database [21]. Computational disease definitions were extracted from the Human Phenotype Ontology [22] (HPO) resource (06/2021 release).

### Variant filtering

SvAnna lets the user filter the input variants by depth of coverage and by population frequency. The depth of coverage is reported in idiosyncratic manner depending on the variant caller. We tested SvAnna to work with three long read structural variant callers: pbsv, sniffles [13], and SVIM [14] (Additional file 1: Table S1). If the required attributes are not present, SvAnna still includes the variant in the output.

For population frequency-based filtering, we considered the variants occurring in more than 1% of the population as common. SVs called in the VCF file are removed from the analysis if they show greater than 80% reciprocal overlap with a common variant in any of the source databases (DGV, gnomAD-SV, dbSNP, and HGSVC SVs). The user can adjust the frequency and reciprocal overlap thresholds via the command line interface.

### Variant prioritization

To prioritize an SV, SvAnna first determines the set of all genes *G* = {*g*_1_, *g*_2_, …}—either the genes with at least one transcript affected by the SV or the closest upstream and downstream genes in case of an intergenic SV. SvAnna applies different rules for the various categories of SV. For each gene *g* ∈ *G*, a sequence deleteriousness score *δ*(*g*) is calculated to reflect the predicted effect of the variant on gene functionality and dosage*,* where *δ* stands for deleteriousness. The rules for calculating *δ*(*g*) differ according to the SV type and are described in the following sections and summarized in Table 1. *δ*(*g*)=0 for presumed neutral variants, with higher scores representing higher degrees of predicted deleteriousness.

**Table 1:** Summary of rules for calculating sequence deleteriousness score *δ*(*g*)

SV class	***t ⊂ SV***	***t*** ⇌ ***SV***	*SV* ⊂ *e*			
			*Coding or splice*	*UTR*	*Intronic*	*Promoter*
**DEL**	1	1	{0.8, 1}	0 ≤ *δ*(*g*)≤1	0	0.4
**DUP**	1^a^	0	{0.8, 1}	0 ≤ *δ*(*g*)≤1	0	0.4
**INV**	0	1	1	0 ≤ *δ*(*g*)≤1	0	0.4
**INS**	–	–	{0.2, 0.9}	0 ≤ *δ*(*g*)≤1	0	0.4
**BND**	–	–	1	1	1	0.4

Higher scores indicate a greater degree of predicted deleterious effect on transcript function. *t* ⊂ *SV*: The SV fully contains the transcript in question. *t* ⇌ *SV*: Partial overlap of the transcript and the SV. *SV* ⊂ *e* The SV is completely contained within the indicated sequence element. {0.8, 1} and {0.2, 0.9} indicate scores for {in-frame, frameshift} variants

^a^Duplication of the entire gene is assigned a score of 1, triplication is assigned a score of 2, and so on

#### Deletions, duplications, and inversions

Scoring *δ*(*g*) for deletions and duplications depends on the type of sequence affected. The maximal deleteriousness is scored for coding sequences. *δ*(*g*)=1 for deletions that disrupt the sequence of a transcript by removing the entire transcript or part of the coding sequence by deletion of one or more exons. For untranslated regions (UTRs), the score *δ*(*g*) for gene *g* is determined as a function of SV and UTR lengths as follows:

1 $$\delta (g)=\mathit{\min}\left(\frac{2\ len_{SV}}{len_{UTR}},1\right)$$

A deletion that encompasses 50% or more of the UTR will be assigned a score of 1 (maximal deleteriousness). Smaller deletions will receive proportionally less deleterious scores.

Analogously, a duplication that adds an entire copy of a transcript without disrupting the coding sequence is assigned a *δ(g)* score of 1 (a triplication would be assigned a score of 2, and so on). A duplication that affects coding sequence and/or splice site regions receives a *δ(g)* score of 1; however, a tandem duplication that extends beyond either start or end of the transcript, and thus does not alter the primary linear transcript sequence, is regarded as neutral (*δ(g)* = 0).

If a breakpoint of an inversion disrupts the coding sequence of a transcript, it is assigned a score of 1. However, an inversion that completely contains a transcript is assumed to be neutral, and receives a score of *δ*(*g*)=0.

#### Insertions

To score insertions located within the coding sequence of a transcript, SvAnna checks if the insertion disrupts the reading frame. Frame-shifting insertions are assigned a *δ(g)* = 0.9 and the insertions that do not alter the reading frame receive *δ(g)* = 0.2. If the insertion is located in the 5′ or 3′ UTR, the *δ(g)* score is determined as a function of insertion and UTR length:

2 $$\delta (g)=\mathit{\min}\left(\frac{len_{INS}}{len_{UTR}},1\right)$$

An insertion that adds the number of bases corresponding to 100% of the UTR or more will be assigned a score of *δ*(*g*)=1. Shorter insertions receive proportionally lower deleteriousness scores. Insertions located outside of the coding sequence, splice site regions, promoter, and UTRs are assigned a score of *δ(g)* = 0 (non-deleterious).

#### Translocations

Translocation breakpoints that disrupt the transcript sequence or the promoter region are assigned a score of *δ*(*G*)=1. The scoring process applies to both breakends of a translocation.

### Phenotype matching

SvAnna takes a list of HPO terms describing the clinical manifestations of the proband as input. It matches them to the 7981 computational disease models of the HPO using symmetric Resnik matching [23]. Briefly, to calculate the Resnik symmetric matching score ɸ*(Q, D)* for disease *D* annotated with *{d*_*1*_*, …, d*_*m*_*}* HPO terms and query *Q* consisting of *{q1, …, q*_*n*_*}* HPO terms describing the clinical manifestations of the proband, SvAnna uses:

3 $$\phi \left(Q,D\right)=\frac{sim\left(Q\to D\right)+ sim\left(D\to Q\right)}{2}$$

where *sim*(*Q* → *D*) is a method for obtaining one-sided semantic similarity between *Q* and *D.* The one-sided semantic similarity is obtained as:

4 $$sim\left(Q\to D\right)=\frac{1}{m}\ {\sum}_{i=0}^m{\mathit{\max}}_{d\in D} IC\left( MICA\left({q}_i,d\right)\right)$$

To save computational time, SvAnna pre-calculates the information content of the most informative common ancestor *IC(MICA(t*_*1*_*, t*_*2*_*))* for all terms *t* used to annotate computational disease models.

### The PSV score

The Pathogenicity of Structural Variation (PSV) score is calculated based on the sequence deleteriousness score *δ(g)* and phenotype similarity score ɸ*(Q, D)* for all affected genes *G*. The sequence score *δ(g)* for each affected gene is weighted by the phenotypic similarity score ɸ*(Q, D)*.

5 $$PSV\left(Q,G,D\right)={\sum}_{g\in G}\delta (g)\cdot {e}^{\phi \left(Q,D\right)}$$

Here, the PSV score is calculated as a function of the query HPO terms (*Q*), the set of affected genes *G*, and the Mendelian diseases *D* associated with the genes in *G*. *δ(g)* is weighted by the exponentiated phenotypic similarity ɸ*(Q, D)* of the query terms *Q* to a computational model of a disease *D* that is associated with variants in *g*. SvAnna uses the highest ɸ*(Q, D)* if more than one disease is associated with variants in *g*.

### Performance benchmarks and comparison of with other algorithms for ranking pathogenic SVs

We conducted a manual review of the scientific literature to create a dataset of disease-associated SVs for benchmarking SvAnna and other tools for SV annotation and prioritization. The dataset comprised 188 disease-associated SVs from 182 case reports published in 146 articles (Table 2, Additional file 1: Fig. S1). In addition to genomic coordinates and genotypes of the causal variants, we recorded NCBI Gene and OMIM identifiers for the causal gene and the associated disease, and we encoded the clinical features of the proband into Human Phenotype Ontology terms [22, 24]. The curated SVs included deletions, duplications, inversions, insertions, and translocations affecting a differing number of genomic elements. The number of clinical features ranged from 1 to 22 with a median of 5 features per case. We recorded the case reports in the Global Alliance for Genomics and Health (GA4GH) Phenopacket format [25]. The phenopackets are available at Zenodo (https://zenodo.org/record/5071267) [26].

**Table 2:** Summary of curated collection of deleterious SVs

	Deletions	Duplications	Inversions	Insertions	Translocations	***Total***
**Multiple genes**	7	2	4	N/A	5	*18*
**Multiple exons**	37	19	5	N/A	N/A	*61*
**Single exon**	81	16	2	3	N/A	*102*
**Promoter**	5	0	0	0	0	*5*
**Intronic**	2	0	0	0	N/A	*2*
***Total***	*132*	*37*	*11*	*3*	*5*	**188**

We curated a collection of 188 published deleterious SVs based on 182 cases published in 146 clinical case reports. We considered five classes of SVs commonly present in LRS variant calling results: deletions, duplications, inversions, insertions, and translocations. We further classified the SVs into five functional categories based on the number of affected genes and the relative location of the SV region with respect to transcripts of genes. The case reports are available for download

To provide a realistic background in the performance benchmark, we used ten VCF files with SVs generated by PacBio long read whole genome sequencing (see below) of peripheral blood samples obtained from ten individuals. The ten individuals are not related to 182 curated case reports. Since all benchmarked tools evaluate each variant independently, we used a simple strategy to simulate a variant dataset by adding disease-associated SVs into a background VCF file derived from PacBio whole-genome sequencing (see below). We ran the benchmark on a case basis; we added all variants of the case report (i.e., both SVs in case of compound heterozygous genotype) in turn to one of ten background VCF files, and we recorded the median SV rank from the ten VCF files. We used the benchmarking schema to compare SvAnna with AnnotSV [27], X-CNV [28], SvScore [29], and ClassifyCNV [30]. The benchmark was performed using the pipeline engine Nextflow [31]. In our performance benchmarks, variants whose ALT allele is supported by < 3 reads are removed prior variant prioritization. To ensure that the causal SV is not filtered out prior to prioritization, we set allelic depth to 5:5 when evaluating heterozygous SV, and 0:10 when evaluating homozygous or hemizygous SV.

#### AnnotSV

*AnnotSV* is an open source tool that annotates structural variants stored in VCF and BED format with functional, regulatory, and clinical information and classifies SVs into five pathogenicity classes: *benign*, *likely benign*, *variant of uncertain significance*, *likely pathogenic*, and *pathogenic*. *AnnotSV* reports results in the tabular format. We performed a local installation of *AnnotSV* v3.0.9 (accessed on June 25 2021). We used *full* annotation mode when running *AnnotSV* to produce a single row per SV in the result file, and we set the *-SVminSize* to 1 to ensure SVs shorter than 50 bp (including causal SVs) are analyzed. The tabular output reports the annotated SVs as one SV per row, and the rows are ordered by decreasing priority. We used the row number to determine the variant rank. If the causal variant was assigned *pathogenicity class = NA* instead of one of the five pathogenicity classes, and therefore missed, we assigned the variant the rank 40,000.

#### SVScore

*SVScore* [29] is a tool for scoring structural variants by aggregating genome-wide CADD [32] scores. The CADD scores are aggregated in multiple ways (operations), and reported to the output VCF file. We used UCSC’s LiftOver tool to remap the variant coordinates into GRCh37 reference genome and we ran *SVScore* v0.6 with the following operations: sum, max, mean, meanweighted, top10, and top10weighted*.* To calculate the variant rank, we extracted values of the SVSCOREMAX, SVSCORESUM, SVSCOREMEAN, and SVSCORETOP10 attributes from the VCF INFO field. If the value was equal to − 1, we assigned the variant the rank 40,000.

#### X-CNV

*X-CNV* [28] uses extreme gradient boosted trees to predict deleteriousness of deletions and duplications in the form of meta-voting prediction (MVP) score. The MVP score integrates several dozens of features, including SV characteristics (type, allele frequency based on DGV, dbVar), functional deleteriousness predictions, non-coding features (CTCF, cCREs, pELS, and dELS elements), and genome-wide annotations. *X-CNV* ingests variant coordinates in BED format and provides a CSV file with feature values and MVP score. To benchmark *X-CNV*, we used UCSC’s LiftOver tool to remap the coordinates of deletions and duplications into GRCh37 reference genome, and we stored the coordinates into a BED file. We ran *X-CNV* (accessed on February 9 2022), and we used the MVP score to determine the variant rank. If the causal variant was not a deletion or duplication, and therefore missed, we assigned the variant the rank 40,000.

#### ClassifyCNV

*ClassifyCNV* [30] is a tool that calculates numeric deleteriousness scores for deletions and duplications, and assigns the ACMG category based on predefined score thresholds. The tool follows the criteria of the ACMG scoring rubrics to assign points, and writes the overlapping dosage-sensitive genes, protein-coding genes, the clinical classification, and the sum of points into a TSV file. To benchmark *ClassifyCNV*, we converted the coordinates of deletions and duplications into BED format and we ran *ClassifyCNV* v1.1.1 with default parameters. We used the *Total score* to determine the variant rank. In case of tied variant scores, the variant was ranked as the variant located in the middle of the tied variant group. We assigned rank 40,000 to all non-deletions and non-duplications.

### Long read sequencing

We used VCF files from ten in-house long read (PacBio) whole genome sequencing experiments as background files for the simulations in order to provide a realistic background in the performance benchmark. Samples were obtained from Ludwig Boltzmann Institute for Rare and Undiagnosed Diseases, Vienna, Austria, the Department of Human Genetics, New York State Institute for Basic Research in Developmental Disabilities, Staten Island, New York, USA, and the Children’s Hospital of Philadelphia with relevant ethical approval. The ten background VCF files are not related to the 182 case reports used for performance benchmarks. We used a similar strategy to assess the performance of the Exomiser tool [33] under the assumption that a more accurate assessment of the accuracy of prioritization results can be obtained with real rather than simulated VCF files, and indeed the performance of Exomiser with in-house VCF files was inferior to the performance obtained by using VCF files from the 1000 Genomes Project Consortium as background [34]. Currently, we are not aware of collections of publicly available long-read VCF files that could be used for bioinformatic simulations.

#### High molecular weight (HMW) DNA extraction

The HMW gDNA was extracted using the Gentra Puregene (Qiagen) kit. Frozen cells or tissues were first pulverized using a mortar and pestle and transferred to a 15 mL tube that contained Qiagen Cell Lysis Solution. The lysate was then incubated with Proteinase K for 3 h at 55 °C, followed by RNase A for another 40 min at 37 °C. Samples were cooled on ice, and Protein Precipitation Solution was added. Samples were then vortexed and centrifuged. The supernatant was transferred to a new tube containing isopropanol for precipitation. Pellet was washed with 70% ethanol, air dried, and rehydrated in PacBio Elution Buffer until dissolved.

#### PacBio HiFi whole genome sequencing

This protocol was carried out using the PacBio SMRTbell Express Template Prep Kit 2.0 and the SMRTbell Enzyme Cleanup Kit. Fifteen micrograms of DNA was sheared to 20 kb using g-TUBE (Covaris). The sheared DNA was purified using Ampure PB beads (PacBio). Ten micrograms of sheared DNA was used in removing single strand overhangs, followed by DNA damage repair and End repair/A-tailing. The repaired/modified DNA was used for V3 adapter Ligation. The adapter ligated library was treated with Enzyme mix for Nuclease treatment to remove damaged or non-intact SMRTbell templates. The purified library was then size selected using two-step size selection with Blue Pippin (Sage Science) generating 9–13 kb and > 15 kb fractions. The size selected and purified > 15 kb fraction of the library was used for sequencing on a Sequel II device.

#### Alignment and variant calling

We aligned long-read PacBio HiFi WGS data to the GRCh38 (hg38) reference using pbmm2 (v1.3.0) with *--preset CCS* option enabled. We identified SVs on the indexed bam files using pbsv (v2.4.0).

## Results

We developed SvAnna, a tool for phenotype-driven annotation and prioritization of SVs detected in LRS. SvAnna was designed to prioritize a broad range of SV classes such as deletions, duplications, inversions, copy number variants (CNVs), insertions, and translocations that affect one or more genes. SvAnna filters out common SVs and calculates a numeric priority score for the remaining rare SVs by integrating information about genes and promoters with phenotype matching to prioritize potential disease-causing variants. SvAnna outputs its results as a comprehensive tabular summary and as an HTML file intended for human consumption that visualizes each variant in the context of affected transcripts, enhancers, repeats, and dosage sensitive regions, providing information about the effects of the variant on transcripts, chromosomal locations, and Mendelian diseases associated with the affected genes.

### Pathogenicity of Structural variation (PSV) score

SvAnna assesses each variant in the context of its genomic location. SvAnna first compares each variant to three sources of common SVs on the basis of reciprocal overlap. In addition to Database of Genomic Variants (DGV), gnomAD-SV, and dbSNP, which are largely based on data from SRS, SvAnna includes a recent dataset of SVs called by LRS (HGSVC) [6]. Common variants are removed according to user-defined frequency and overlap constraints (Methods).

For each SV, SvAnna determines the extent of overlap with genomic elements, including promoters and transcripts. For each transcript, it determines which exon or exons are affected and whether the transcriptional start site of the coding sequence is disrupted.

**Fig. 1:**
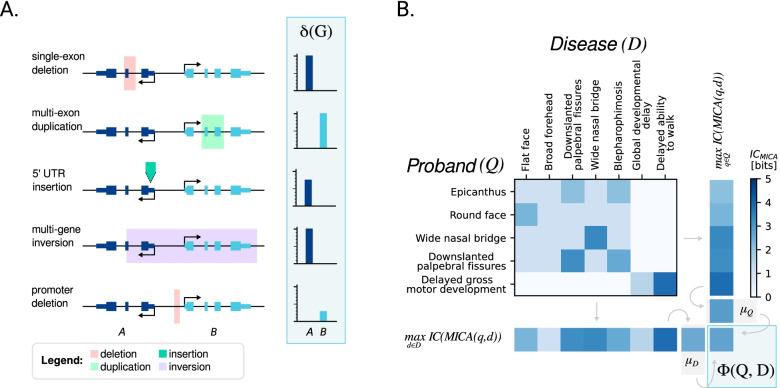
Overview of SvAnna algorithm. **A** Sequence deleteriousness score *δ(G)*. The score assesses deleteriousness (predicted effect on gene function) by means of a series of heuristics for different SV classes (Table 1). **B** Phenotype similarity score *Φ(Q,D)*. SvAnna calculates the phenotypic similarity for a set of HPO terms *Q* representing the patient’s phenotypic features and HPO terms *D* for a disease. SvAnna computes the information content (IC) of the most informative common ancestor (MICA) for all term pairs *q*, *d* for *q* ∈ *Q *and﻿* d* ∈ *D*. The mean ICs *μ*_*Q*_ and *μ*_*D*_ are calculated for Q and D, and the final similarity score *Φ* is calculated as the mean of *μ*_*D*_ and *μ*_*Q*_. The *δ(G)* and the *Φ(Q,D)* scores are combined to obtain the final PSV score (Methods)

For each class of variant, SvAnna defines rules to assess a sequence deleteriousness score *δ*(*G*) for a set of genes *G* affected by the variant (Fig. 1A). At the same time, a phenotypic relevance score *Φ(Q,D)* is calculated based on the similarity of patient phenotypes *Q* encoded using Human Phenotype Ontology [22, 24] (HPO) terms and the ~ 8000 computational disease models *D* of the HPO project (Fig. 1B). The candidates are ranked based on a PSV score that is calculated as a function of the *δ(G)* and *Φ(Q,D)* scores. The following sections explain the approach to specific classes of SV.

#### Deletions and duplications

To calculate the priority of a deletion of a genomic region, SvAnna determines the relative location of each overlapping gene with respect to the variant. Deletions can fully contain a transcript, partially overlap, or be contained in a transcript region. SvAnna assigns complete transcript deletions a *δ(g)* score of 1. Deletions of single exons are assigned a score of 1 if they include coding sequence or a canonical splice site region and lead to shift of the reading frame. In-frame deletions involving one or more exons receive a score of 0.8. If a deletion is located in an intron, it is assigned a score of 0 (not deleterious). If a deletion affects an untranslated region (UTR), it is assigned a score based on the length of the SV and the UTR, with higher (more deleterious) scores being assigned to SVs that are large compared to the UTR sequence. In some cases, the effect of an SV is different for different transcripts of a gene. SvAnna chooses the highest transcript score and uses that score as the *δ(g)* score for the gene. If a deletion encompasses multiple genes, the *δ(g)* score is assigned in this way for each gene.

**Fig. 2:**
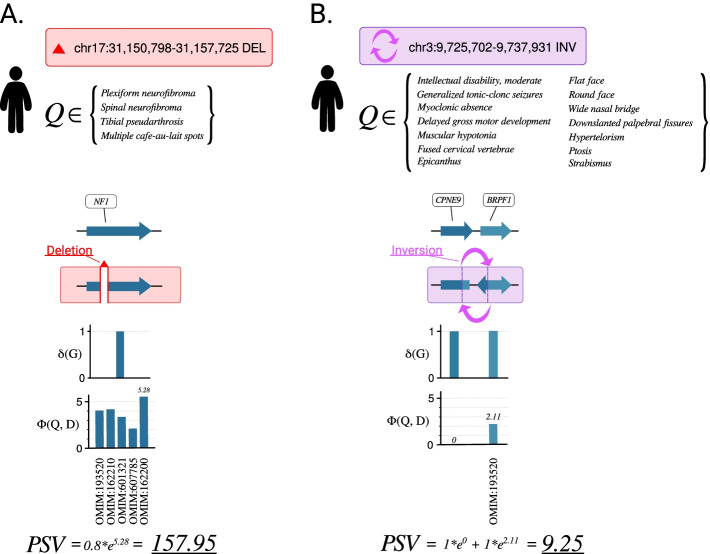
Prioritization of variants. **A** A case of proband with a single-exon deletion in the *NF1* gene [35]. The deletion results in *δ(g) = 0.8* for *NF1*. To calculate semantic similarity *Φ(Q,D)* for *NF1,* SvAnna evaluates five computational disease models associated with variants in *NF1*. In case of this proband, *Neurofibromatosis, Type I* (OMIM:162200) is the disease model that matches the proband’s clinical condition the best (*Φ(Q,D)* = 5.28). As *NF1* is the only gene affected by the deletion, *δ(g)* and *Φ(Q,D)* of *NF1* are the only determinants of the final PSV score. **B** A case of proband with an inversion involving 3′ end of *CPNE9* and 5′ end of *BRPF1* [36]. SvAnna assigns *δ(g)* score of 1 to both *CPNE9* and *BRPF1* that are disrupted by the inversion. Unlike the case of *NF1* variant, the inversion involves > 1 genes; therefore, the final PSV integrates the scores of phenotypically relevant *BRPF1* (8.25) and disrupted, but phenotypically non-relevant *CPNE9* (1.00)

For example, a ~ 6.9 kb-long deletion that leads to in-frame loss of 48 amino-acid residues encoded by exon 2 of *NF1* (NM_000267.3) is considered to alter the function of the gene (*δ(g) = 0.8*) as the deletion removes the entire exon from the transcript [35]. Together with the phenotype features of the proband consisting of *plexiform neurofibroma* (HP:0009732), *spinal neurofibromas* (HP:0009735), *tibial pseudarthrosis* (HP:0009736), and *multiple cafe-au-lait spots* (HP:0007565), the variant attains rank 1 with a final PSV score of 157.95 (Fig. 2A). SvAnna generates an HTML output of the variant and its position with respect to the affected transcripts, repetitive elements, and enhancers (see Additional file 1: Fig. S2 for this example). The median rank of 81 cases with single exon deletions was 1.

A deletion of three exons in *BRCA1* received a PSV score of 427.0 and rank 1 (Additional file 1: Fig. S3). The median rank of multiple-exon deletions was 1. SvAnna uses a slightly different approach to prioritize multigene SVs. For instance, a deletion at chr2:109,923,337–110,405,062 (hg38) affects four genes (*LIMS3*, *MALL*, *NPHP1*, and *MTLN*). SvAnna calculates the *δ(g)* score of each gene as 1, weighted by the phenotype score according to equation 5 (Methods). Only *NPHP1* is associated with a phenotypically relevant disease (Joubert syndrome 4) and its contribution to the final PSV score is highest (Additional file 1: Fig. S4). The median rank of deletions affecting multiple genes was 1.

Duplications are handled in a similar way to deletions except that the gene *g* that is entirely spanned by a duplication is assigned *a δ(g)* score of 1. Similar considerations about duplications that affect individual exons or an entire transcript pertain as with deletions. Tandem duplications that do not alter the primary linear sequence of a transcript (e.g., a duplication of the final exon of a transcript) are assigned a score of 0 (i.e., are assumed not to be deleterious). For example, a pathogenic duplication of 36 bp within one exon of the *PIBF1* gene was assigned a PSV score of 3.38 (Additional file 1: Fig. S5).

#### Inversions

An inversion is prioritized using a similar approach to that used for evaluating a deletion, with several differences. The *δ(g)* score for a genomic element spanned by an inversion is defined as 0 (since the primary sequence of the element is unchanged). If the sequence of a transcript is interrupted by inversion breakends, a *δ(g)* score of 1 is assigned. Additionally, inversions that affect one or multiple (but not all) exons of a transcript are assigned a score of 1. An inversion located completely within an intron is assigned a score of 0 (not deleterious).

For example, breakpoints of a 12 kb copy neutral inversion identified in monozygotic twins suffering from intellectual disability disrupt genic regions of *BRPF1* and *CPNE9* [36]. Since each breakpoint disrupts the gene sequence, the *δ(g)* score is set to *1* for both genes. Deleterious variants in *BRPF1* are associated with *Intellectual developmental disorder with dysmorphic facies and ptosis* (OMIM:617333). The final PSV score of 9.25 aggregates scores of two disrupted genes: phenotypically relevant *BRPF1* (8.25) and disrupted, but phenotypically not relevant *CPNE9* (1.0) (Figs. 2B, Additional file 1: Fig. S6).

#### 5′UTR and transcriptional start site variants

SvAnna has specific rules for prioritizing SVs in non-coding sequences. SvAnna assumes that variants affecting UTR regions, especially SVs, are less likely to have a functional impact on gene expression or translation. SvAnna calculates the *δ(g)* score for a UTR variant as a function of the variant length and UTR length (Methods). However, the variants that disrupt transcription start sites (TSS) are considered just as deleterious as variants that affect coding sequences. As an example, we evaluated de novo deletion of 1571 bp affecting the first non-coding exon of *AMER1* [37]. The deletion was processed as a loss of TSS, leading to a PSV score of 10.4 (Additional file 1: Fig. S7).

#### Promoter variants

SvAnna extends the prioritization rules to variants in gene promoters with a potential to change the gene expression. The promoter regions are assumed to encompass 2 kb upstream of the TSS. To calculate the *δ(g)* score, SvAnna assigns SVs in promoter regions a score that is 40% of that of an SV in a coding sequence. Effectively, this means that only promoter variants in genes with a good phenotype match get high priority scores. This is a limitation of the approach that could be overcome as our ability to build computational models of promoter variants improves. If the variant affects a promoter and another genic region (e.g., TSS, UTR), the most deleterious *δ(g)* score for any of the regions is used to calculate the PSV score. For example, a 13-bp deletion in the promoter of the von Willebrand factor (*VWF*) gene in a patient with type 1 von Willebrand disease [38] was assigned a PSV score of 63.2 (Additional file 1: Fig. S8).

#### Translocations

SvAnna applies a series of rules to assess the pathogenicity of translocations. A translocation that disrupts the coding sequence of a gene *g* or separates the transcription start site of *g* from its coding sequence is assigned a *δ(g)* score of 1. The PSV score is calculated based on the predicted effects of the translocation at both breakpoints. For example, a translocation that disrupts the coding sequence of *SLC6A1* in a case of myoclonic-atonic epilepsy was assigned a PSV score of 4.74 (Additional file 1: Fig. S9).

#### SvAnna achieves clinically relevant sensitivity

**Fig. 3:**
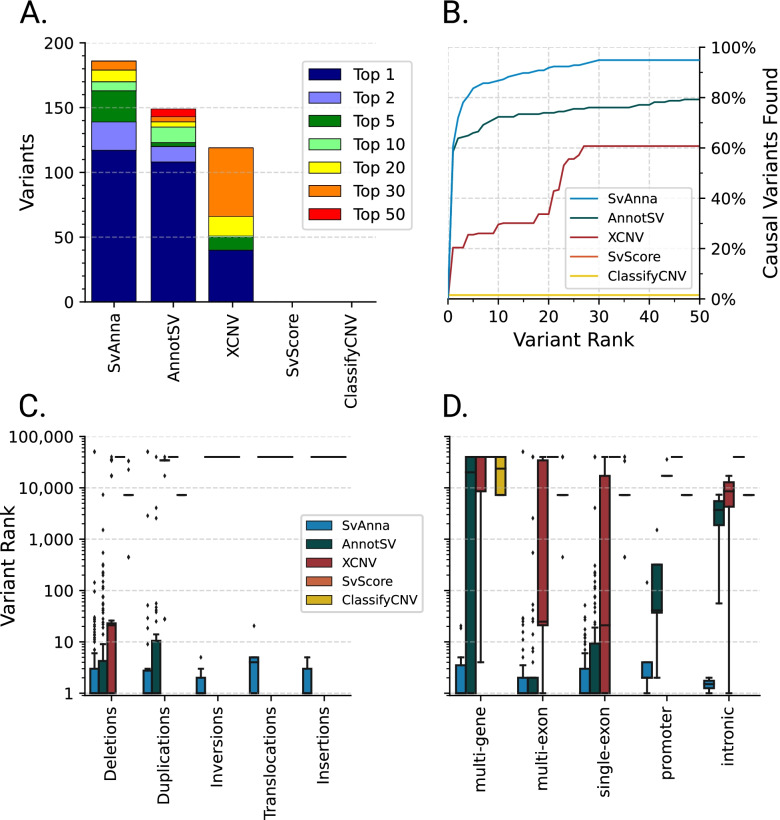
Comparison of prioritization performance of different methods for prioritization of SVs. **A** Median ranks of 188 deleterious SVs obtained from simulated analysis runs. Top 5 means that the rank assigned by the tool was between 1 and 5, and so on. **B** Plot showing the cumulative rank for prioritizations by SvAnna, AnnotSV, X-CNV, SvScore, and ClassifyCNV. **C** SvAnna assigns the best rankings to all 5 evaluated SV classes. **D** SvAnna attains the best median ranks for SVs of all sizes, performing notably well in prioritization of variants involving multiple genes. In **C** and **D**, the boxes represent distributions of the median ranks. Each box plot is defined so that the center line is at the median variant rank, the box borders mark the 25th and 75th percentiles, and the whiskers stretch to denote 1.5 times the interquartile range

We are not aware of any other tool that specifically targets VCF data as produced by modern LRS SV callers. We ran SvAnna on 10 in house VCF files called with pbsv, sniffles [13], and SVIM [14] and were able to annotate ~ 99.9% of variants (i.e., identify nature and position of overlaps with transcripts). We are aware of only one comparable published tool for VCF-based phenotype-driven SV prioritization: *AnnotSV* [27], a standalone command-line script that annotates SVs with functional, regulatory, and clinical information to filter out neutral variants and rank the candidate pathogenic variants, while the phenotype matching is delegated to a separate tool, Exomiser [33]. *AnnotSV* is able to annotate only DEL and DUP calls and missed INS, BND, CNV, and INV calls that were processed by SvAnna. For instance, on a VCF file called by pbsv, AnnotSV missed 25,198 (40%) of 63,084 variants. We additionally tested three representative tools for SV analysis; we transformed the coordinates of CNVs found in the ten LRS VCF files into BED format to enable comparison with SVScore, X-CNV, and ClassifyCNV. SvAnna showed substantially superior performance to these tools (Fig. 3).

No other tool we are aware of is able to process all SV categories found in typical long-read VCF files as SvAnna can.

To assess the practical utility of SvAnna and to compare performance with *AnnotSV*, *SVScore*, *X-CNV*, and *ClassifyCNV*, we developed a simulation strategy based on 182 curated case reports (Methods). We used 10 VCF (62,337–107,233 variants) generated by our in-house LRS pipeline and added the causal variant(s) to simulate 10 runs per curated case, for a total of 1,820 data sets. Then, we prioritized the simulated variant dataset and calculated the median rank for the causal variant across 10 runs. Overall, SvAnna placed the causal variant on the top of all variants in 60% of cases, the causal variant was at rank 10 or better in 87% of cases, and 91% of variants were placed at a rank of 20 or better (Fig. 3A, B).

We further evaluated the performance on different variant types. SvAnna showed consistent performance for all variant types from our benchmark set. SvAnna was confident in prioritization of deletions, duplications, inversions, and insertions, assigning median variant rank of 1. The breakend variants had a median variant rank of 4. SvAnna supports prioritization of all variant types (Fig. 3C, D). SvAnna performed consistently in cases with both low and high numbers of HPO clinical features (Additional file 1: Fig. S10).

#### SvAnna software

**Fig. 4:**
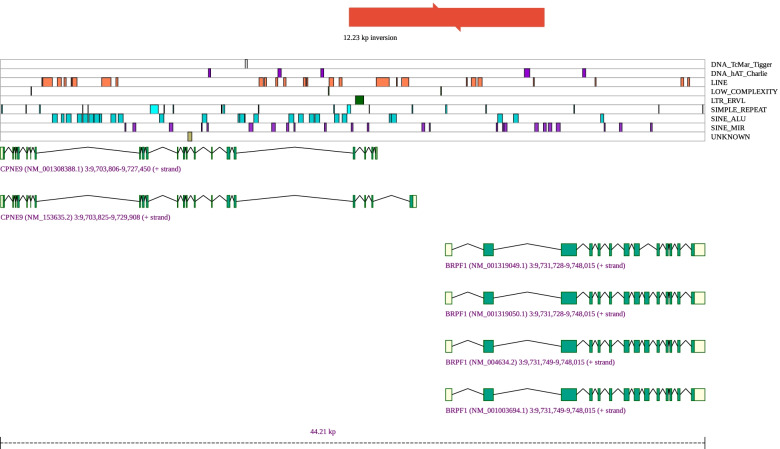
Inversion affecting *BRPF1*. Screenshot of the graphic generated by SvAnna for inv(chr3)(9725702; 9737931), a ∼12.23 kb inversion that disrupts the coding sequence of the *CPNE9* and *BRPF1* genes observed in patient with intellectual disability with dysmorphic features [36]. The graphic displays the relative location of the inversion (red box) with respect to individual transcripts of the affected genes. The transcripts are drawn as boxes (exons) and lines (introns) where green represents the coding regions, and yellow the non-coding regions. In addition, the graphic presents nearby repeat sequence loci to help with discovering variant calling artifacts, as well as interpretation of deleterious SVs that are often flanked with repeat regions

SvAnna presents its results both as tabular and VCF files suitable for bioinformatics analysis as well as a visually appealing HTML report to support clinical interpretation. The HTML report is a single page with a tabular and graphical summary of the top 100 variants, with information about the variant (read counts, VCF id, position and length, and genotype if available), genes that overlap the variant and Mendelian diseases associated with the genes [39], and a list of all overlapping transcripts as well as the position and effect on the transcript. A graphical display is generated for each variant (such as those shown in Fig. 4 and Additional file 1: Figs. S2-S9) as a scalable vector graphics (SVG) file that is embedded directly in the HTML code. The SVG shows the SV and its position compared to that of overlapping transcripts, whose coding exons are shown in green and non-coding exons in yellow. If applicable, overlapping repeats [21] and VISTA enhancers [20] are shown as tracks beneath the variant (Screenshot in Additional file 1: Fig. S11). The SVGs additionally display dosage sensitive regions (haploinsufficiency and triplosensitivity) as reported by ClinGen [40]. SvAnna is made freely available for academic use as a Java command-line application. The application annotates a VCF file containing ~ 60,000 SVs in ~ 3 min on a consumer laptop. The GitHub repository contains source code, a pre-built executable, and links to detailed instructions for use, as well as a VCF file with the eight examples presented here and a tutorial.

## Discussion

Our ability to analyze the role of SVs in Mendelian disease has lagged significantly behind as compared to our capability to interpret single nucleotide variants and other small variants. The reasons for this include technical difficulties in calling SVs, the relative lack of functional data on the effects of SVs on gene regulation, and the paucity of genome-wide association studies for SVs [1]. The advent of LRS promises to greatly improve the detection of SVs in patient samples. PacBio LRS was shown to be about three time more sensitive than a SRS ensemble calling approach, with the improvement was predominantly derived from improved detection of repeat-associated SV classes, particularly of intermediate-sized SVs (50 bp to 2 kb), and insertions across the SV size spectrum [41]. However, progress on many fronts will be required to fully realize the promise of LRS for genetic medicine, including continued technical improvements, cost reductions, better SV calling algorithms, and more comprehensive knowledge of the medical relevance of classes of SVs that were difficult or impossible to ascertain with previous technologies.

Compared to the large variety of approaches available for SRS, there are very few computational methods for assessing the relevance of SVs for rare disease [5]. Numerous algorithms, databases, and tools have been developed to support the medical interpretation of diagnostic SRS. Although details vary from tool to tool, in general, variant pathogenicity is assessed on the basis of variant allele population frequencies, evolutionary conservation, and functional impact prediction for missense, splice, and regulatory variants [42–44]. Disease genes can be prioritized based on functional and genomic data [45], or on the basis of phenotypic similarity of patient phenotype definitions with computational disease models of the HPO project [22, 46].

There is a need to extend these algorithms for LRS. SvAnna includes a number of innovations to this end. VCF files represent SVs in multiple ways including the default (sequence) representation and symbolic notation. Translocations are represented as breakend calls on two lines. SvAnna uses a harmonized computational model of variants to represent each category of variant, which enables it to apply a single prioritization approach to all categories of SV. We are aware of only one previously published tool for phenotype-driven prioritization of SVs, AnnotSV [27]. In contrast to SvAnna, AnnotSV was primarily designed to analyze SV events identified in SRS and array-based experiments and only supports the analysis of deletions and duplications. SvAnna demonstrated a substantially better overall performance than AnnotSV in the ranking of all classes of the causal SVs. SVScore, X-CNV, and ClassifyCNV do not include phenotype matching into the variant scoring. Furthermore, X-CNV and ClassifyCNV are limited to deletions and duplications. These factors may partially explain their relatively poorer performance compared to SvAnna.

In our study, SvAnna prioritized 87% of SVs in the first 10 ranks. The case reports were chosen from 182 publications, seven of which reported diagnostic results from LRS [4, 10, 12, 36, 47–49]. Current published experience with LRS in a human genetic diagnostic setting is limited, and it is too early to assess the potential advantage of LRS over SRS in diagnostic settings. Given that LRS detects SVs in genomic regions that were difficult or impossible to characterize by SRS, the medical relevance of variation in these regions will need to be assessed. SvAnna will benefit from future updates to the HPO resource in this area.

SvAnna’s ability to predict the medical relevance of SVs that affect presumptive enhancer sequences is limited. We are aware of no current database with a comprehensive representation of Mendelian-disease associated SVs in regulatory sequences, and the majority of relevant case reports in the literature are not linked to enhancer databases such as VISTA or FANTOM. As information accrues in the literature about mechanisms of disease-associated variation in enhancer sequences, it will be important to develop predictive models to accelerate discoveries.

SvAnna runs in < 5 min for a typical genome on a consumer laptop (faster if computations are performed on multiple threads). All required data for running SvAnna are provided as a compressed archive for download. SvAnna is implemented as a standalone application with no external dependencies.

## Conclusions

We developed SvAnna, an interpretable method for phenotype-driven prioritization of deleterious SVs obtained from high-throughput sequencing experiments. SvAnna is currently the only tool for phenotype-based prioritization of SVs that is specifically designed to work with VCF files produced by typical LRS SV callers. We are likely to be at the beginning of a period of rapid expansion of LRS in diagnostic settings. SvAnna will play an important role in this process by promoting improved clinical interpretation of a range of SVs. The interpretable prioritizations provided by SvAnna will facilitate the widespread adoption of LRS in diagnostic genomics. SvAnna is freely available for academic use at https://github.com/TheJacksonLaboratory/SvAnna [15].

## Supplementary Information

**Additional file 1: Table S1.** Summary of VCF fields used to report depth of coverage. **Fig. S1.** Distribution of lengths of the curated structural variants. **Figs. S2-S9.** Examples of graphical displays generated for different types of structural variants. **Fig. S10.** Mean variant rank depending on the number of phenotype terms. **Fig. S11.** Screenshot of tabular and graphical summary generated for structural variants.

## Data Availability

SvAnna source code is freely available for academic use on GitHub (https://github.com/TheJacksonLaboratory/SvAnna) [15]. SvAnna pre-compiled executable JAR file along with database files are available for download via the SvAnna documentation site (https://svanna.readthedocs.io/en/master). Svart is available at its GitHub site (https://github.com/exomiser/svart) [16]. The 182 case reports of 188 deleterious SVs are available for download from Zenodo (https://zenodo.org/record/5071267) [26]. The case reports are in Global Alliance for Genomics and Health (GA4GH) Phenopacket format [50]. Consent was not obtained for sharing the raw data such as the sequence reads or the variant call format files from the 10 probands. The datasets of common SVs are available at the following locations: Database of Genomic variants (DGV): [17], GnomAD SV: https://ftp.ncbi.nlm.nih.gov/pub/dbVar/data/Homo_sapiens/by_study/genotype/nstd166/gnomad_v2.1_sv.sites.vcf.gz, HGSVC SV: http://ftp.1000genomes.ebi.ac.uk/vol1/ftp/data_collections/HGSVC2/release/v1.0/integrated_callset/freeze3.sv.alt.vcf.gz, dbSNP: https://ftp.ncbi.nih.gov/snp/organisms/human_9606_b151_GRCh38p7/VCF/00-common_all.vcf.gz. pbmm2 is available at https://github.com/PacificBiosciences/pbmm2. pbsv is available at https://github.com/PacificBiosciences/pbsv.
