# Supplementary material for: SvAnna: efficient and accurate pathogenicity prediction of coding and regulatory structural variants in long-read genome sequencing
Source: Genome Med. 2022 Apr 28;14:44. doi: 10.1186/s13073-022-01046-6 (PMC9047340; doi:10.1186/s13073-022-01046-6)
Supplement: Supplementary file 1 — Additional file 1: Table S1. Summary of VCF fields used to report depth of coverage. Fig. S1. Distribution of lengths of the curated structural variants. Figs. S2-S9. Examples of graphical displays generated for different types of structural variants. Fig. S10. Mean variant rank depending on the number of phenotype terms. Fig. S11. Screenshot of tabular and graphical summary generated for structural variants. [file 13073_2022_1046_MOESM1_ESM.pdf]

**Table S1. Summary of VCF fileds used to report depth of coverage**. SvAnna uses variant zygosity, and the number of reads that support presence of the reference and alternate alleles (allelic depth) in the analysis. Due to flexibility of the VCF format, allelic depth can be reported in an idiosyncratic manner. We tested SvAnna with variant calls from three long read structural variant callers: pbsv, Sniffles, and svim.

| Variant caller | Attribute | VCF tag |
|---|---|---|
| pbsv | # ref reads | AD |
| pbsv | # alt reads | AD |
| Sniffles | # ref reads | DR |
| Sniffles | # alt reads | DV |
| svim | # ref reads | AD |
| svim | # alt reads | AD |

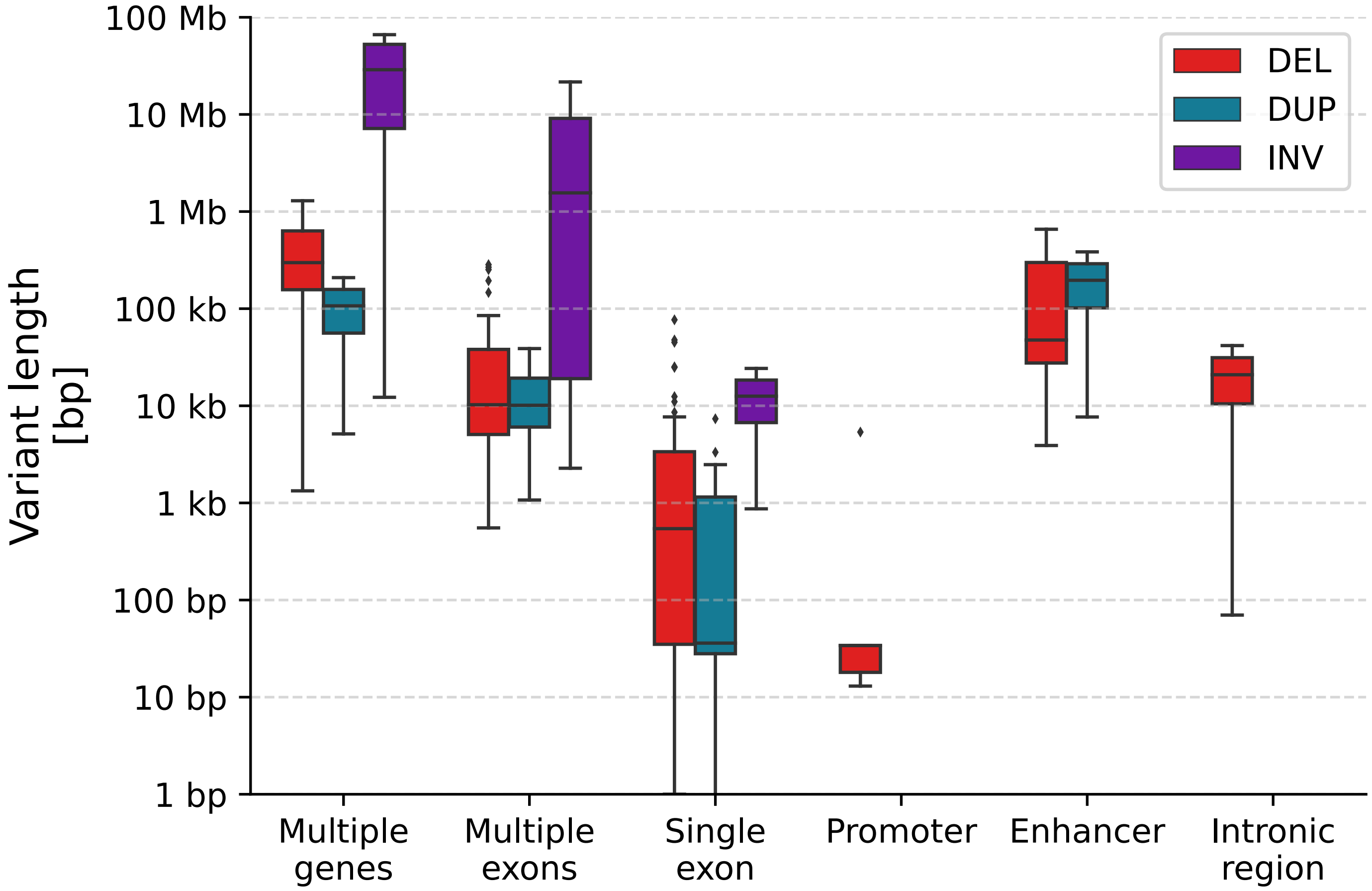


**Fig. S1. Distribution of lengths of the curated structural variants**. Insertions and translocations are considered to have zero length (not shown).

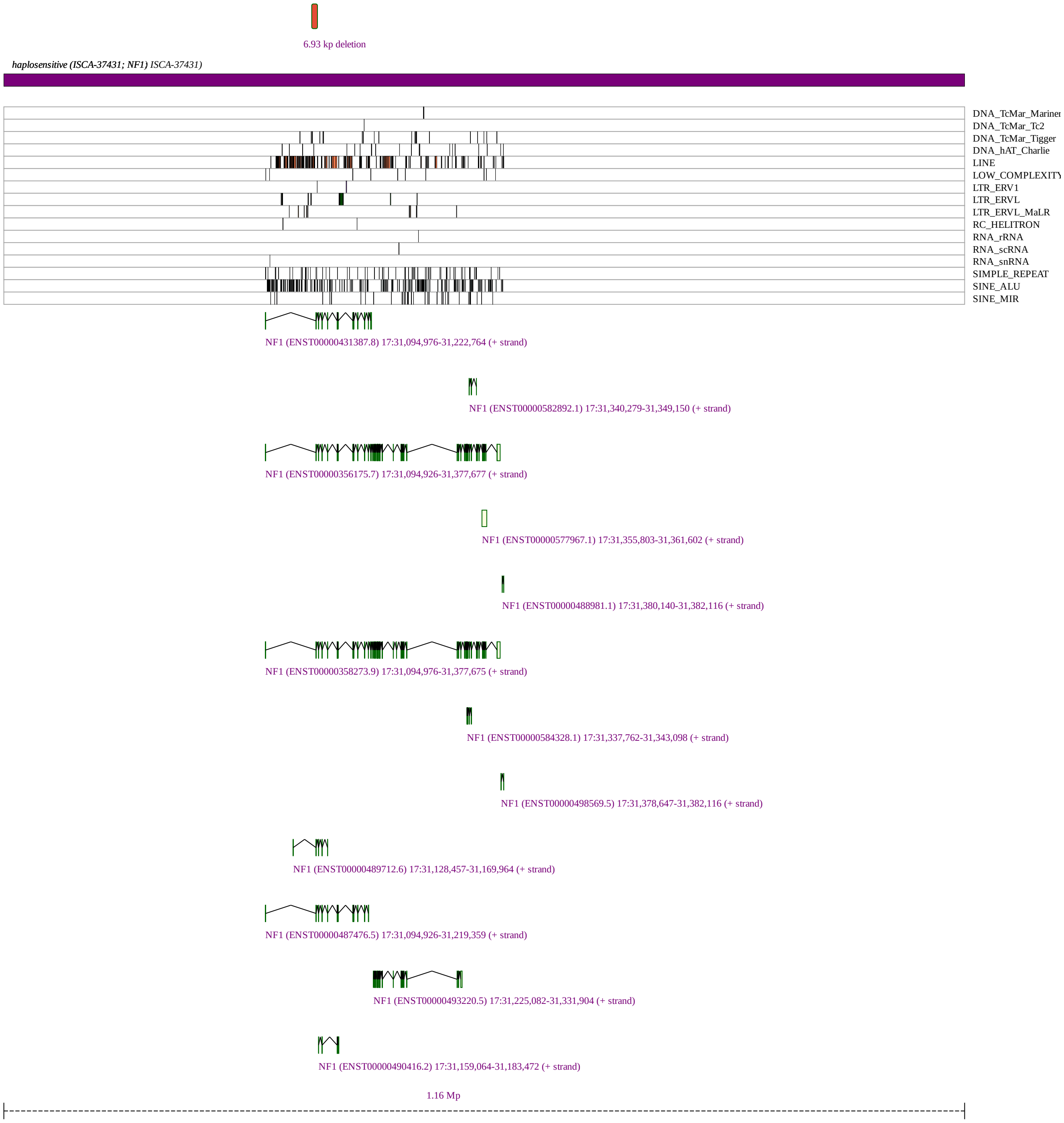


**Fig. S2. Deletion affecting** *NF1*. Screenshot of the graphic generated by SvAnna for chr17:31,150,798-31,157,725del, a deletion of 6.93 kb that was assigned a PSV score of 157.95. The deletion affects exon 2 of several *NF1* transcripts. Pathogenic variants in *NF1* are associated with neurofibromatosis type 1 (OMIM:162200). The phenotypic features curated for this case were *Multiple cafe-au-lait spots* (HP:0007565), *Plexiform neurofibroma* (HP:0009732), *Spinal neurofibromas* (HP:0009735), and *Tibial pseudarthrosis* (HP:0009736). Data were curated from a published case report [1].

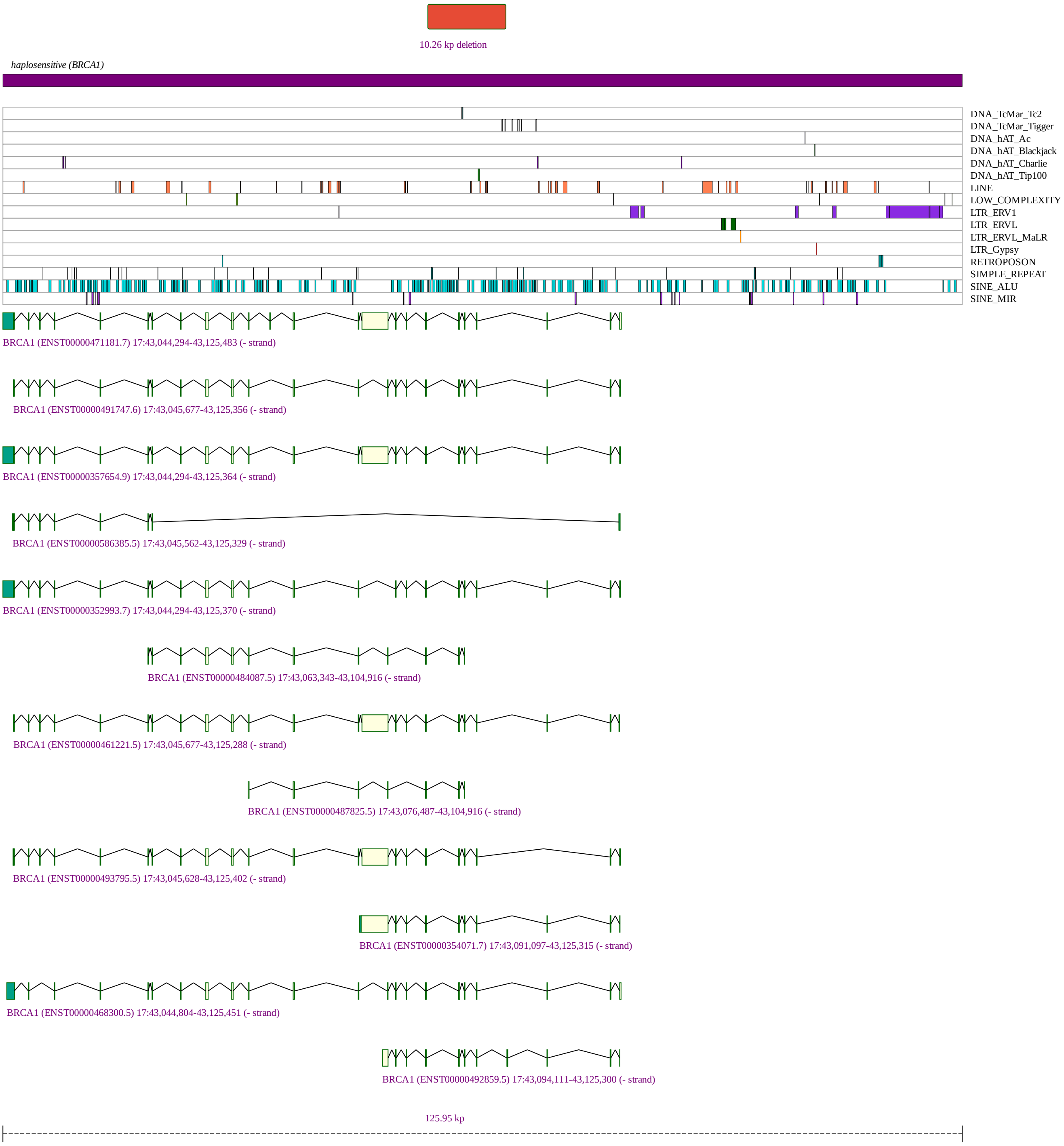


**Fig. S3. Deletion affecting** *BRCA1*. Screenshot of the graphic generated by SvAnna for chr17:43,100,079-43,110,335del, a deletion of 10.26 kb that was assigned a PSV score of 427.00. The deletion affects three *BRCA1* exons. Pathogenic variants in *BRCA1* are associated with Breast-ovarian cancer, familial, 1 (OMIM:604370). The phenotypic feature curated for this case was *Breast carcinoma* (HP:0003002). Data were curated from a published case report [2].

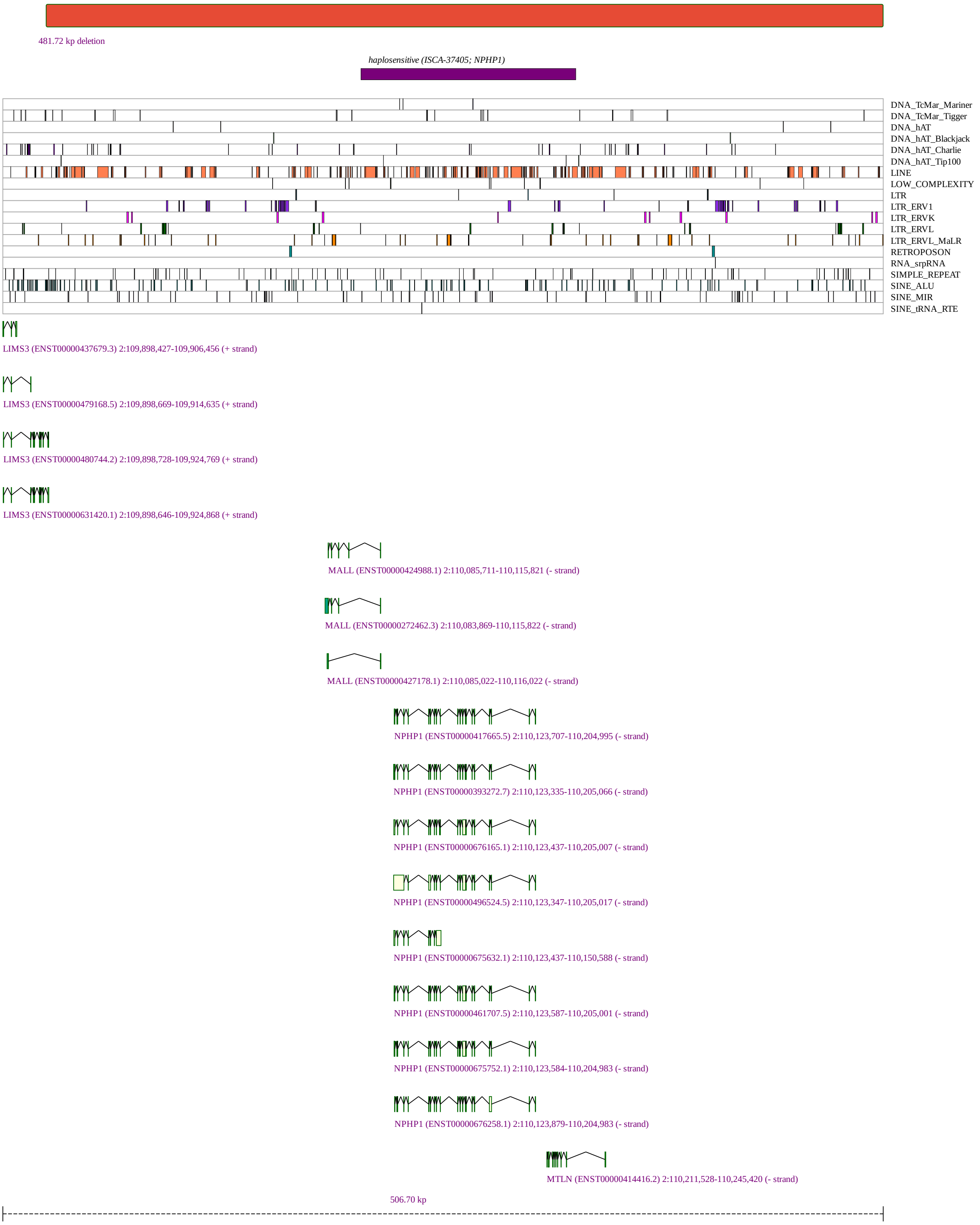


**Fig. S4. Deletion affecting** *NPHP1*. Screenshot of the graphic generated by SvAnna for chr2:109,923,337-110,405,062del, a deletion of 481.73 kb that was assigned a PSV score of 17.60. The deletion is predicted to disrupt *MALL*, *NPHP1*, and *MTLN*. Pathogenic variants in *NPHP1* are associated with Joubert syndrome 4 (OMIM:609583). The phenotypic features curated for this case were *Stage 5 chronic kidney disease* (HP:0003774), *Cerebellar vermis hypoplasia* (HP:0001320), *Truncal ataxia* (HP:0002078), *Blindness* (HP:0000618), *Ptosis* (HP:0000508), *Molar tooth sign on MRI* (HP:0002419), *Elongated superior cerebellar peduncle* (HP:0011933), *Limb ataxia* (HP:0002070), *Optic disc pallor*(HP:0000543), and *Coloboma* (HP:0000589). Data were curated from a published case report [3].

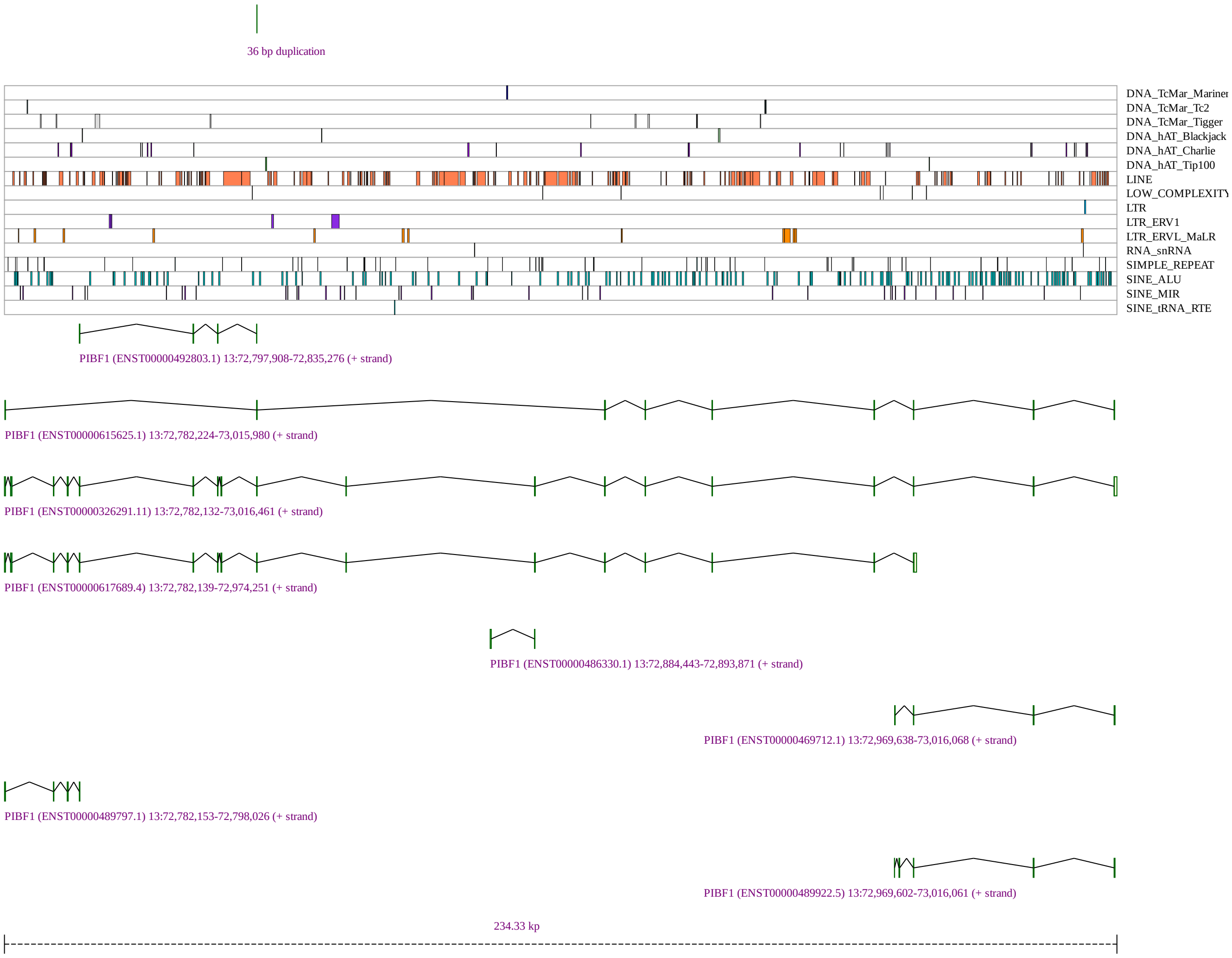


**Fig. S5. Duplication affecting** *PIBF1.* Screenshot of the graphic generated by SvAnna for `chr13:72835296-72835332dup`, a duplication of 36 bp that was assigned a PSV score of 3.38. Pathogenic variants in PIBF1 are associated with *Joubert syndrome 33* (OMIM:617767). The phenotypic features curated for this case were *Periglomerular fibrosis* (HP:0032417), *Vesicoureteral reflux* (HP:0000076), *Hypoplasia of the corpus callosum* (HP:0002079), *Ascites* (HP:0001541), *Hypermetropia* (HP:0000540), *Feeding difficulties* (HP:0011968), *Seizure* (HP:0001250), *Deeply set eye* (HP:0000490), *Global developmental delay* (HP:0001263), *Areflexia* (HP:0001284), *Hepatomegaly* (HP:0002240), *Generalized hypotonia* (HP:0001290), *Hyaline casts* (HP:0031200), *Midface retrusion* (HP:0011800), *Nephronophthisis* (HP:0000090), *Renal tubular atrophy* (HP:0000092), *Acute kidney injury* (HP:0001919), *Perisylvian polymicrogyria* (HP:0012650), *Molar tooth sign on MRI* (HP:0002419), *Ventriculomegaly* (HP:0002119), and *Enlarged kidney* (HP:0000105). Data were curated from a published case report [4].

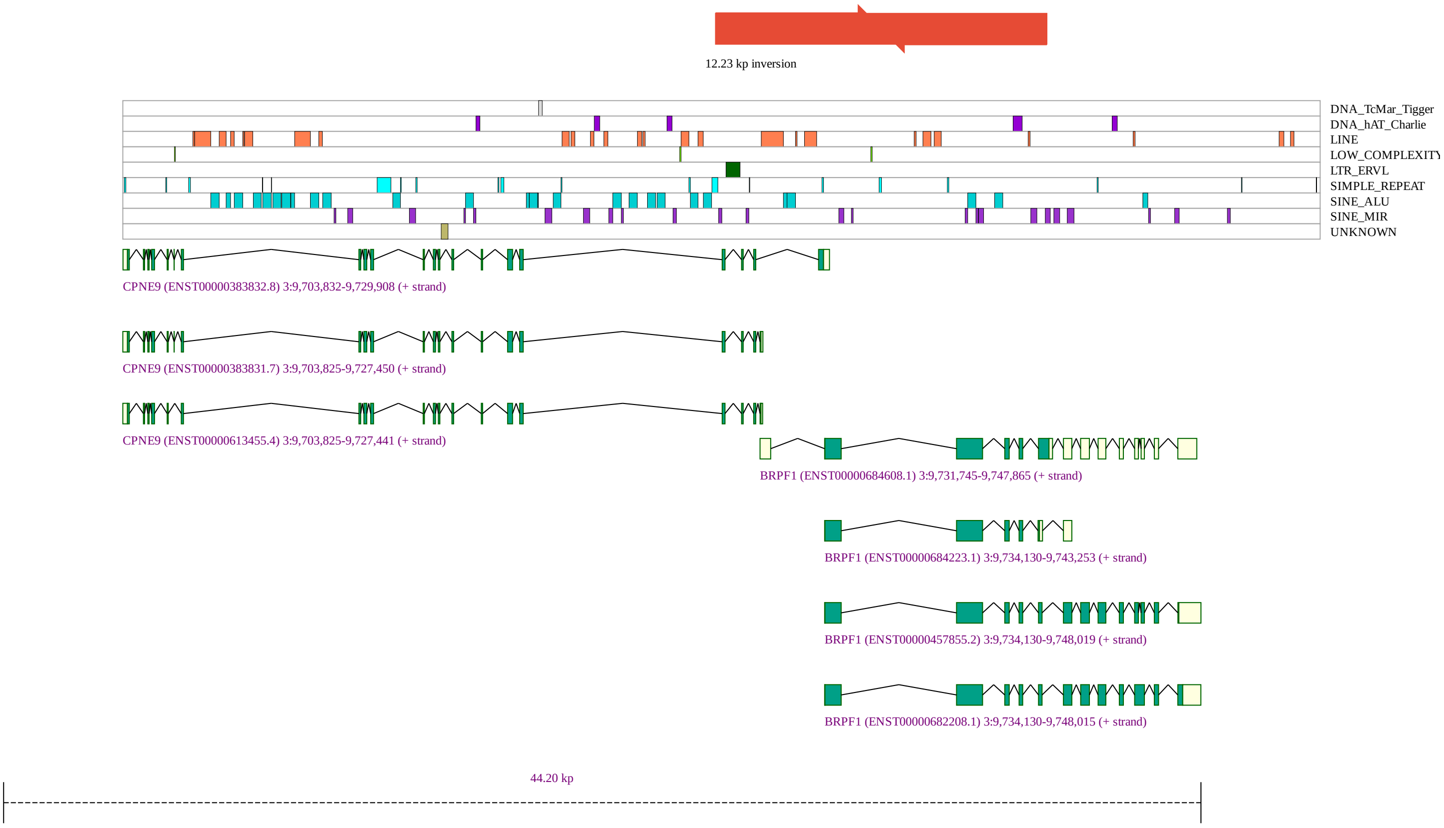


**Fig. S6. Inversion affecting** *BRPF1*. Screenshot of the graphic generated by SvAnna for `inv(chr3)(9725702; 9737931)`, a ∼12.23 kb inversion that disrupts the coding sequence of the *BRPF1* gene with a PSV score of 9.25. Pathogenic variants in *BRPF1* are associated with *Intellectual developmental disorder with dysmorphic facies and ptosis* (OMIM:617333). The phenotypic features curated for this case were *Epicanthus* (HP:0000286), *Bilateral tonic-clonic seizure* (HP:0002069), *Downslanted palpebral fissures* (HP:0000494), *Intellectual disability, moderate* (HP:0002342), *Strabismus* (HP:0000486), *Delayed speech and language development* (HP:0000750), *Wide nasal bridge* (HP:0000431), *Hypotonia* (HP:0001252), *Delayed gross motor development* (HP:0002194), *Flat face* (HP:0012368), *Myoclonic absence seizure* (HP:0011150), *Fused cervical vertebrae* (HP:0002949), *Ptosis* (HP:0000508), *Hypertelorism* (HP:0000316), and *Round face* (HP:0000311). Data were curated from a published case report [5].

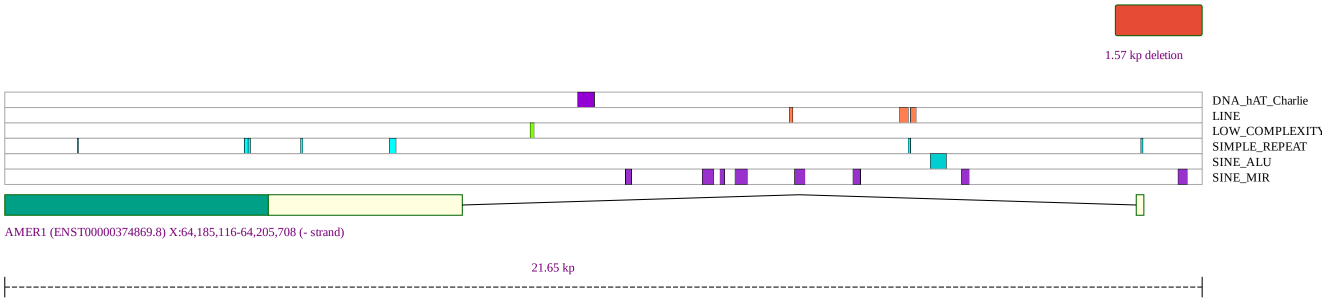


**Fig. S7. Deletion affecting** *AMER1*. Screenshot of the graphic generated by SvAnna for chrX:64,205,190-64,206,761del, a hemizygous deletion of ∼1.57 kb that deletes the transcription start site of the *AMER1* gene and was assigned a PSV score of 10.39. Pathogenic variants in *AMER1* are associated with Osteopathia striata with cranial sclerosis (OMIM:300373). The phenotypic features curated for this case were *Polyhydramnios* (HP:0001561), *Delayed speech and language development* (HP:0000750), *Thickened calvaria* (HP:0002684), *Upper airway obstruction* (HP:0002781), *Hypertelorism* (HP:0000316), *Metaphyseal striations* (HP:0031367), *Bilateral cleft lip and palate* (HP:0002744), *Macrocephaly* (HP:0000256), and *Lymphedema* (HP:0001004). Data were curated from a published case report [6].

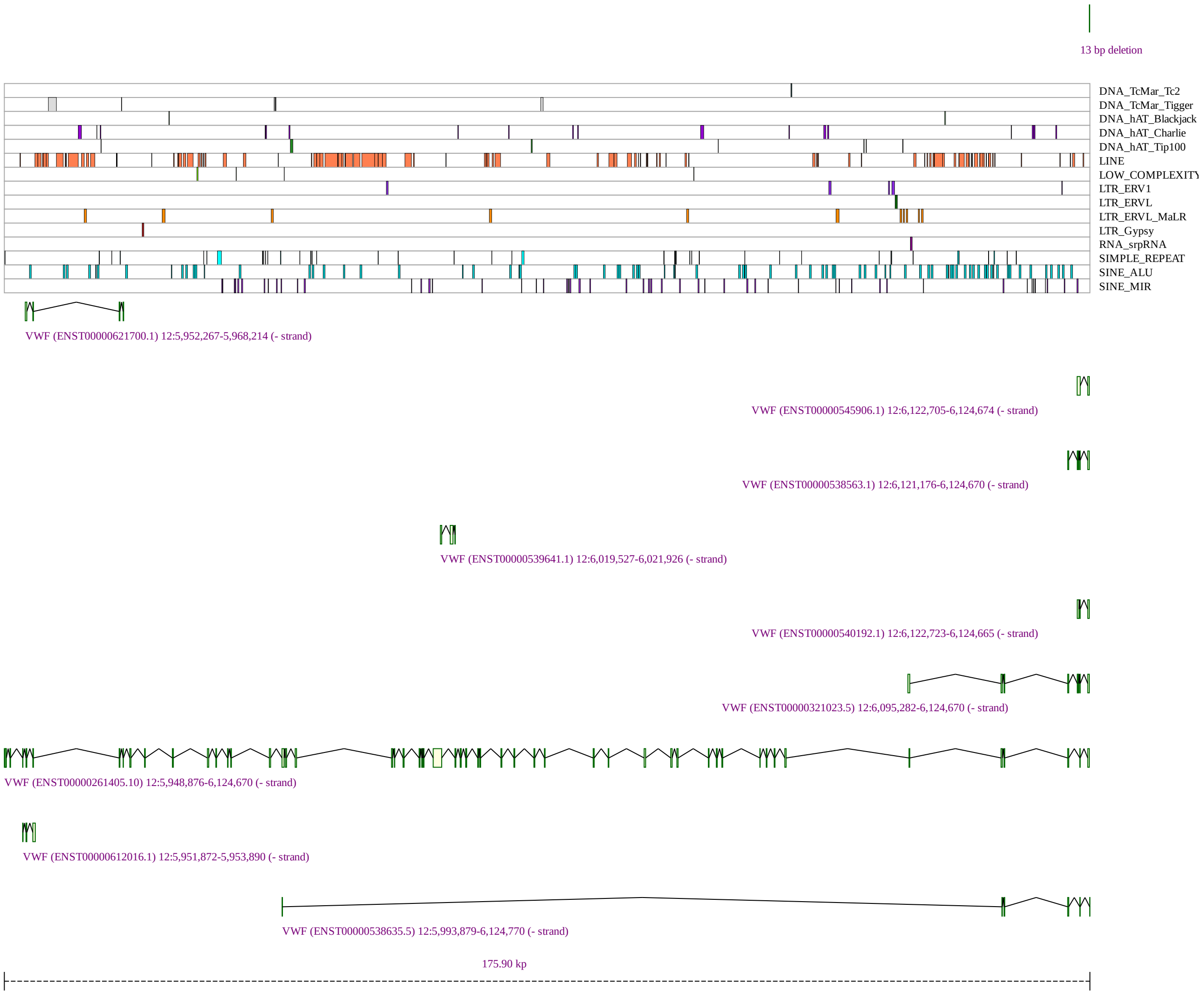


**Fig. S8. Deletion affecting** *VWF*. Screenshot of the graphic generated by SvAnna for chr12:6,124,705-6,124,718del, a 13 bp deletion located in the core promoter region of *VWF* (30 bp upstream of ENST00000261405.10). In the original publication, the deletion was shown to lead to aberrant binding of Ets transcription factors to the site of the deletion and thereby reduce VWF expression [7]. Pathogenic variants in *VWF* are associated with von Willebrand disease (OMIM:193400). The phenotypic features curated for this case were *Prolonged bleeding following procedure* (HP:0011890), *Bruising susceptibility* (HP:0000978), and *Reduced quantity of Von Willebrand factor* (HP:0012147). Data were curated from a published case report [7].

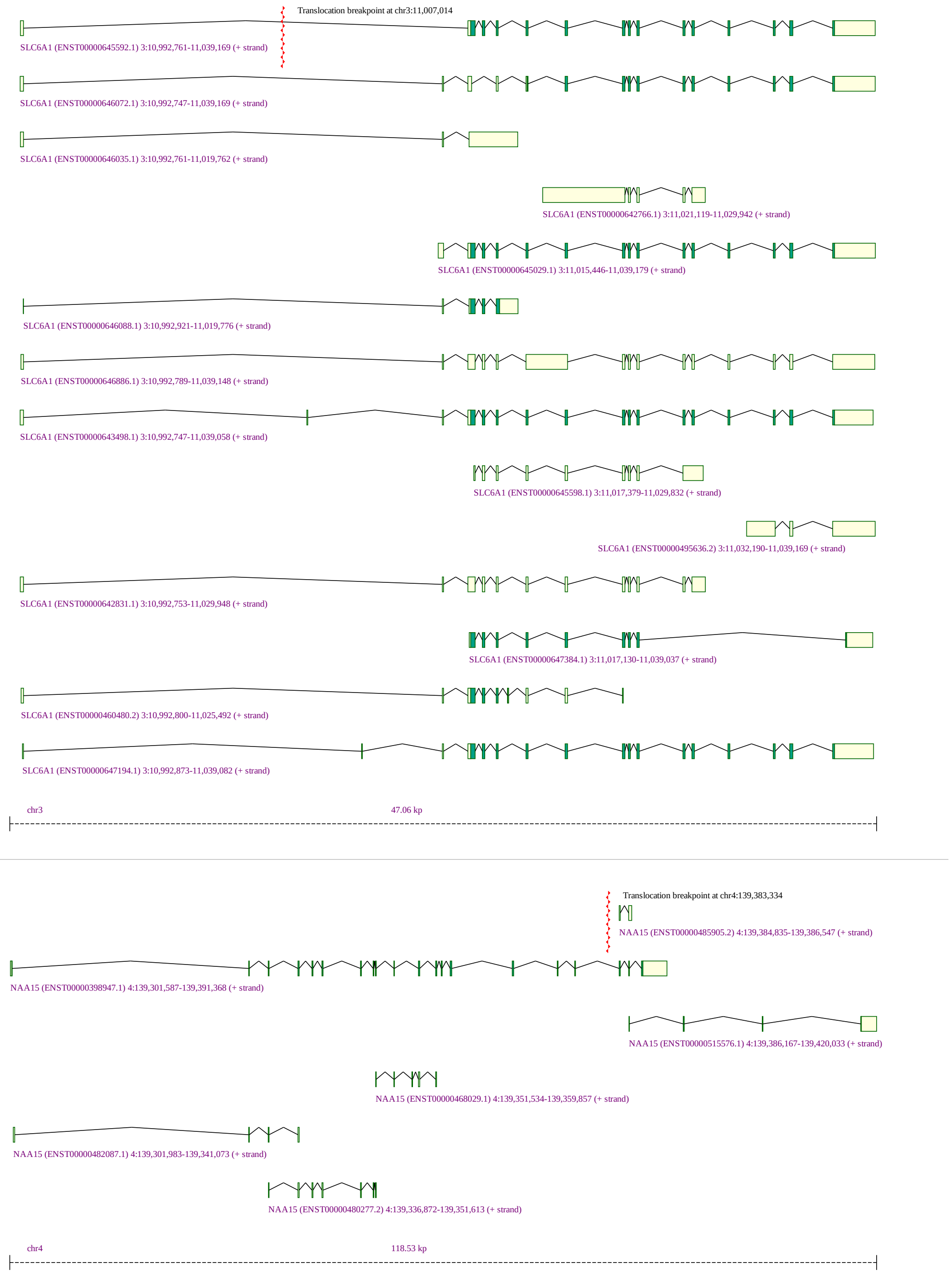


**Fig. S9. Translocation affecting** *SLC6A1*. Screenshot of the graphic generated by SvAnna for `t(chr3:11,007,014; chr4:139,383,334)`, a translocation that was assigned a PSV score of 4.74. Pathogenic variants in *SLC6A1* are associated with *Myoclonic-atonic epilepsy* (OMIM:616421). The phenotypic features curated for this case were *Hypertonia* (HP:0001276), *Microcephaly* (HP:0000252), *Knee flexion contracture* (HP:0006380), *Scoliosis* (HP:0002650), *Seizure* (HP:0001250), *Global developmental delay* (HP:0001263), *Malar flattening* (HP:0000272), *Thin upper lip vermilion* (HP:0000219), *Thick lower lip vermilion* (HP:0000179), *Elbow flexion contracture* (HP:0002987), *Narrow nasal bridge* (HP:0000446). Data were curated from a published case report [8].

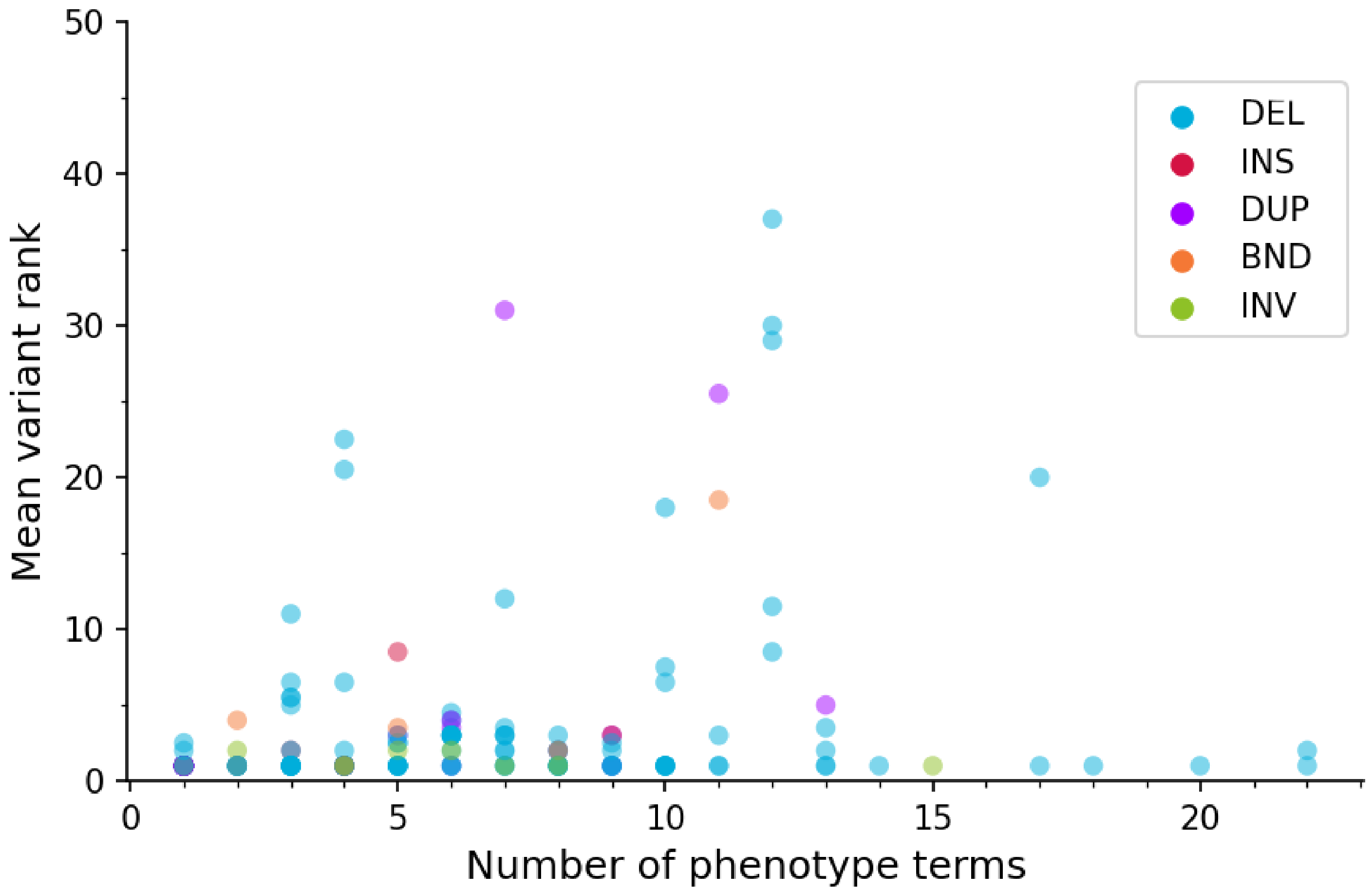


**Fig. S10. Mean variant rank depending on the number of phenotype terms**. The number of phenotype terms ranged from 1 to 22 with median of 5 terms for a case. 5 variants received rank greater than 50 (not shown).

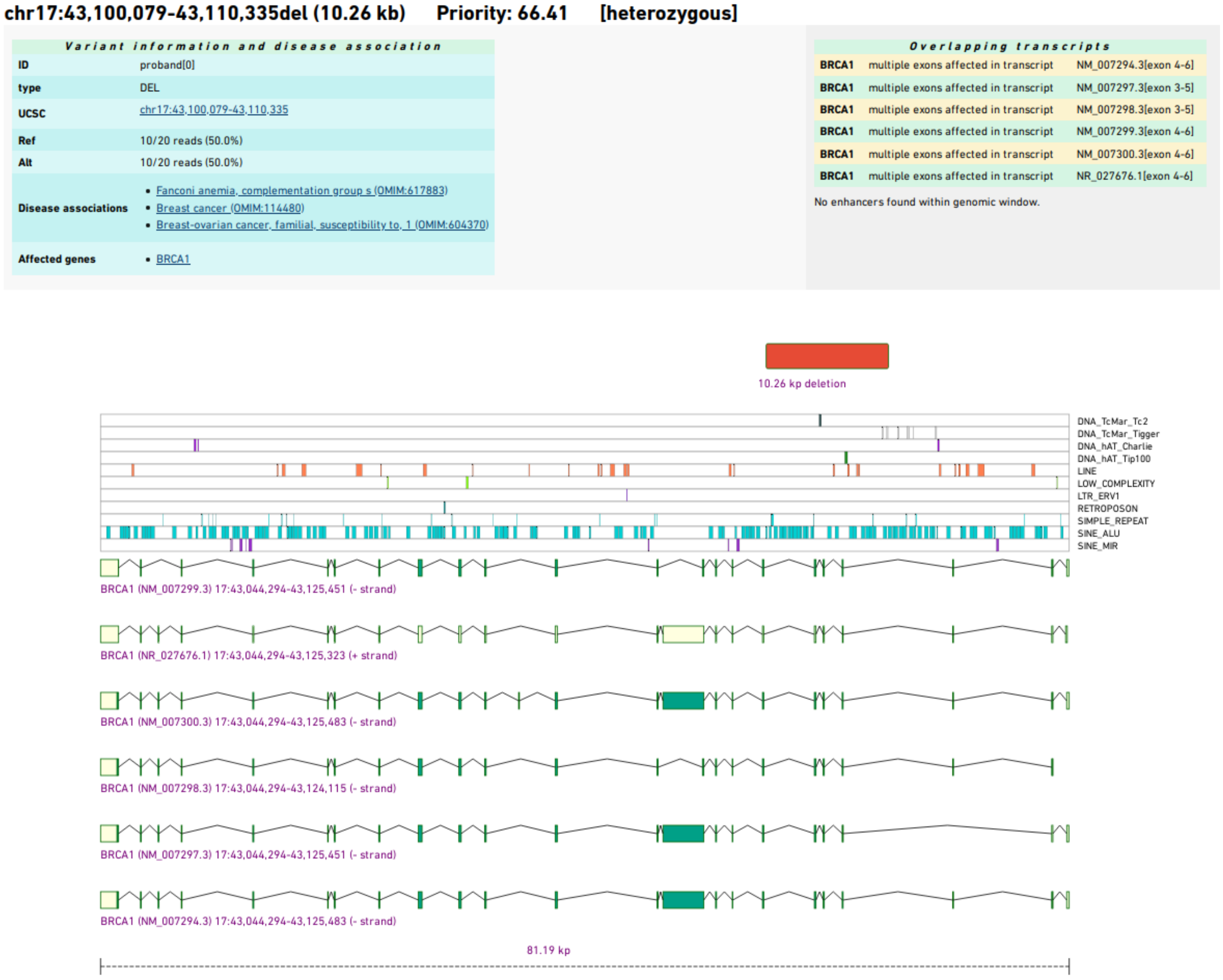

**chr17:43,100,079-43,110,335del (10.26 kb)  Priority: 66.41  [heterozygous]**

| Variant information and disease association | |
|---|---|
| ID | proband[0] |
| type | DEL |
| UCSC | chr17:43,100,079-43,110,335 |
| Ref | 10/20 reads (50.0%) |
| Alt | 10/20 reads (50.0%) |
| Disease associations | • Fanconi anemia, complementation group s (OMIM:617883)<br>• Breast cancer (OMIM:114480)<br>• Breast-ovarian cancer, familial, susceptibility to, 1 (OMIM:604370) |
| Affected genes | • BRCA1 |

| Overlapping transcripts | | |
|---|---|---|
| BRCA1 | multiple exons affected in transcript | NM_007294.3[exon 4-6] |
| BRCA1 | multiple exons affected in transcript | NM_007297.3[exon 3-5] |
| BRCA1 | multiple exons affected in transcript | NM_007298.3[exon 3-5] |
| BRCA1 | multiple exons affected in transcript | NM_007299.3[exon 4-6] |
| BRCA1 | multiple exons affected in transcript | NM_007300.3[exon 4-6] |
| BRCA1 | multiple exons affected in transcript | NR_027676.1[exon 4-6] |

No enhancers found within genomic window.



**Fig. S11.** SvAnna screenshot. SvAnna generates a tabular and graphical summary of the top 100 variants (the number can be adjusted by the user). The chromosomal position, length, PSV score, genotype are listed at the top of each entry. The *Variant information and disease association* table summarizes read count information and the ID of the variant in the VCF file, and provides links to genes affected by the SV and diseases associated with the gene in the Online Mendelian Inheritance in Man (OMIM) resource [9]. The *Overlapping transcripts* table shows each of the transcripts that overlaps with the SV as well as the position and effect on the transcript. The graphical display is generated as a scalar vector graphics (SVG) file that is embedded directly in the HTML code. It shows the SV and its position compared to that of overlapping transcripts, whose coding exons are shown in green and non-coding exons in yellow. If applicable, overlapping repeats and dosage sensitive regions are shown as tracks beneath the variant. In some cases, this may provide a clue as to whether a given SV event could be related to neighboring repetitive sequences. Information for this track is derived from the UCSC Genome Browser database [10].

# References


[1] Meng-Chang Hsiao, Arkadiusz Piotrowski, Tom Callens, Chuanhua Fu, Katharina Wimmer, Kathleen B. M. Claes, and Ludwine Messiaen. Decoding nf1 intragenic copy-number variations. *American journal of human genetics*, 97:238–249, August 2015.

[2] Vesna Musani, Ilona Sušac, Petar Ozretić, Domagoj Eljuga, and Sonja Levanat. The first case report of a large deletion of the brca1 gene in croatia: A case report. *Medicine*, 96:e8667, December 2017.

[3] Shingo Koyama, Hidenori Sato, Manabu Wada, Toru Kawanami, Mitsuru Emi, and Takeo Kato. Whole-exome sequencing and digital pcr identified a novel compound heterozygous mutation in the nphp1 gene in a case of joubert syndrome and related disorders. *BMC medical genetics*, 18:37, March 2017.

[4] Malavika Hebbar, Anil Kanthi, Anju Shukla, Stephanie Bielas, and Katta M. Girisha. A biallelic 36-bp insertion in pibf1 is associated with joubert syndrome. *Journal of human genetics*, 63:935–939, July 2018.

[5] Takeshi Mizuguchi, Nobuhiko Okamoto, Keiko Yanagihara, Satoko Miyatake, Yuri Uchiyama, Naomi Tsuchida, Kohei Hamanaka, Atsushi Fujita, Noriko Miyake, and Naomichi Matsumoto. Pathogenic 12-kb copy-neutral inversion in syndromic intellectual disability identified by high-fidelity long-read sequencing. *Genomics*, 113:1044–1053, January 2021.

[6] J. Mi, P. Parthasarathy, B. J. Halliday, T. Morgan, J. Dean, M. J. M. Nowaczyk, D. Markie, S. P. Robertson, and E. M. Wade. Genes (Basel)Deletion of Exon 1 in AMER1 in Osteopathia Striata with Cranial Sclerosis. *Genes (Basel)*, 11(12), 11 2020.

[7] Maha Othman, Yvette Chirinian, Christine Brown, Colleen Notley, Nicholas Hickson, Daniel Hampshire, Suzanne Buckley, Simon Waddington, Alan L. Parker, Andrew Baker, Paula James, and David Lillicrap. Functional characterization of a 13-bp deletion (c.-1522_-1510del13) in the promoter of the von willebrand factor gene in type 1 von willebrand disease. *Blood*, 116:3645–3652, November 2010.

[8] Karolina Pesz, Victor Murcia Pienkowski, Agnieszka Pollak, Piotr Gasperowicz, Maciej Sykulski, Joanna Kosińska, Magdalena Kiszko, Bogusława Krzykwa, Magdalena Bartnik-Głaska, Beata Nowakowska, Małgorzata Rydzanicz, Maria Małgorzata Sasiadek, and Rafał Płoski. Phenotypic consequences of gene disruption by a balanced de novo translocation involving slc6a1 and naa15. *European journal of medical genetics*, 61:596–601, October 2018.

[9] Joanna S. Amberger, Carol A. Bocchini, Alan F. Scott, and Ada Hamosh. Omim.org: leveraging knowledge across phenotype-gene relationships. *Nucleic acids research*, 47:D1038–D1043, January 2019.

[10] Jairo Navarro Gonzalez, Ann S. Zweig, Matthew L. Speir, Daniel Schmelter, Kate R. Rosenbloom, Brian J. Raney, Conner C. Powell, Luis R. Nassar, Nathan D. Maulding, Christopher M. Lee, Brian T. Lee, Angie S. Hinrichs, Alastair C. Fyfe, Jason D. Fernandes, Mark Diekhans, Hiram Clawson, Jonathan Casper, Anna Benet-Pagès, Galt P. Barber, David Haussler, Robert M. Kuhn, Maximilian Haeussler, and W. James Kent. The ucsc genome browser database: 2021 update. *Nucleic acids research*, 49:D1046–D1057, January 2021.
